# Impact of virtual reality hypnosedation on perioperative pain and anxiety in port implantation under local anesthesia: a randomized controlled pilot trial (VIP Trial)

**DOI:** 10.1186/s13741-024-00454-z

**Published:** 2024-10-10

**Authors:** Kira C. Steinkraus, Hannah Feldmann, Lisa S. Hunold, Sandra Graf, Colette Dörr-Harim, Nadir Nasir, Christoph W. Michalski, Felix J. Hüttner

**Affiliations:** 1 Department of General and Visceral Surgery, Ulm University Hospital, Ulm, Germany; 2 Clinical Trial Centre, Department of Surgery ulmCARES, Ulm University Hospital, Ulm, Germany; 3 Present address: Department of General, Visceral and Transplantation Surgery, University of Heidelberg, Heidelberg, Germany; 4 Present address: Department of General, Visceral and Thoracic Surgery, Klinikum Nürnberg, Paracelsus Medical University, Nuremberg, Germany

**Keywords:** Virtual reality, Hypnosedation, Port implantation, Local anesthesia, Perioperative anxiety, Outpatient surgery

## Abstract

**Background:**

This monocentric randomized controlled pilot trial investigates the impact of virtual reality (VR) hypnosedation on perioperative anxiety, pain, patient satisfaction, and medication usage during port implantation under local anesthesia.

**Methods:**

A total of 120 patients undergoing elective port implantation between January 2022 and August 2023 were enrolled and randomized in a 1:1 ratio to either a VR hypnosedation group or a control group. The VR group used a commercially available VR headset with the HypnoVR application, providing various environments, musical backgrounds, and a guiding voice, while the control group underwent the procedure without VR. Patients with ASA > 3, chronic pain, cognitive issues, and contraindications against VR use were excluded. The main outcomes measured were perioperative pain and anxiety scores, with secondary outcomes including perioperative medication usage. Due to the nature of the interventions, blinding of patients and physicians was not feasible. Statistical analysis was primarily descriptive and exploratory, focusing on estimating effect sizes for future trials.

**Results:**

The study found no significant differences in immediate postoperative pain with 1.43 ± 1.63 vs. 1.6 ± 2.05 (*p* = 0.62) or anxiety scores 30.65 ± 9.13 vs. 31.78 ± 13.34 (*p* = 0.60) between the no VR and VR group, respectively. Additionally, there was a trend to less usage of certain medications, particularly remifentanil (mean dose of 200 mg vs. 100 mg (*p* = 0.12)) and novaminsulfon (mean dose of 1250 mg vs. 900 mg (*p* = 0.26)) in the VR group vs. no VR group, respectively. However, these differences were not statistically significant and therefore no definitive conclusions can be drawn regarding medication usage based on this data.

**Conclusion:**

While VR hypnosedation did not significantly reduce perioperative pain or anxiety in this pilot trial, the observed trends in reduced medication usage suggest potential benefits. These findings warrant further investigation in larger, confirmatory trials to better understand the role of VR in enhancing patient comfort and potentially reducing reliance on pharmacological interventions during surgical procedures.

**Trial registration:**

German Clinical Trials Register: DRKS00028508; registration date 15 March 2022; Universal Trial Number: U1111-1275–4995.

## Background

In recent years, a noteworthy uptrend in outpatient surgical procedures was observed. Among these, intravenous access port implantation, typically carried out under local anesthesia (LA), has gained significant popularity. This shift can be attributed to multiple advantages associated with the use of LA, which include savings in material and manpower, a diminished risk of adverse reactions, cost-effectiveness, and expedited patient discharge compared to procedures that utilize general anesthesia (GA) (Liu et al. 2005; Feo et al. 2017).

However, while the benefits of LA are manifold, it is not without challenges. A primary concern associated with the omission of GA is the heightened perioperative stress and anxiety patients may experience in the operative room setting. Such emotional and psychological distress can be detrimental to postoperative recovery, potentially delaying healing and overall recuperation (Schuld et al. 2009; Stamenkovic et al. 2018).

In the pursuit of alleviating these concerns, emerging technological interventions such as virtual reality (VR) have been explored. VR has demonstrated potential in diminishing pain, anxiety, and emotional discomfort in various medical settings (L. de A. P. Cacau,, et al. 2013; Ericsen 2016; Garrett et al. 2018).

Specifically, studies have highlighted that the application of VR distraction during procedures conducted under LA can lead to appreciable reductions in both pain and anxiety (L. de A. P. Cacau,, et al. 2013; Garrett et al. 2018; Hoxhallari et al. 2019; Andrew et al. 2021; Chan et al. 2018; Dreesmann et al. 2022; Chan and Scharf 2017).

Given this backdrop, the VIP Trial has been conceptualized. The aim of this pilot trial was to ascertain if VR hypnosedation during port implantation under LA has an impact on perioperative anxiety, pain, patient satisfaction, tolerability of the procedure, and perioperative medication usage.

## Materials and methods

### Trial design

This study was designed as a monocentric, randomized controlled pilot trial with two parallel intervention groups and a 1:1 allocation. Participants were deemed eligible if they were undergoing the implantation procedure without the use of general anesthesia.

### Trial population and eligibility criteria

Adult patients undergoing port implantation under LA for any given underlying disease were the target population of the trial. Exclusion criteria were set to ensure patient safety and trial integrity. Individuals were excluded if they had an American Society of Anesthesiologists (ASA) physical status classification > 3. Other exclusion criteria included the presence of chronic pain, cognitive capacity issues prohibiting to follow the VR procedure, uncontrolled epilepsy, auditory or visual disorders, and pre-existing implanted devices (e.g., hearing aids or cardiac pacemakers/defibrillators). Additionally, patients participating in other trials that might interfere with this study’s outcomes or those with significant language barriers that could impede informed consent or understanding of procedure instructions were also excluded.

The sample size had been estimated as sufficient for generating initial efficacy data, enabling a reliable sample size calculation for a subsequent confirmatory trial. This estimation was based on the rules of thumb for sample size estimation in pilot trials as proposed by Whitehead et al. (Whitehead et al. 2016). It was determined that even with a relatively small standardized difference (δ ≤ 0.1), a sample of 50 or more patients per group would suffice to calculate a reliable sample size for a subsequent confirmatory trial with 80% power.

### Intervention/trial-specific procedures

Patients scheduled for elective port implantation for any given indication were assessed for suitability during their outpatient visit. After obtaining consent for the surgical procedure, patients were informed about the trial and were asked for participation. The primary method for port implantation was venae sectio of the cephalic vein. If this was unsuccessful, a modified Seldinger’s technique was used through the cephalic vein. Only when this failed a sonography-guided puncture of the subclavian vein was performed and the port was implanted in Seldinger’s technique as outlined in the PORTAS-3 study (Hüttner et al. 2019). Typically, the port was placed on the patient’s non-dominant side (e.g., left side for right-handed individuals) unless specific factors dictated otherwise. A certified commercial port system was employed with a regular port catheter size of 8 French, or 6.6 French in patients with narrow veins.

In the operating room, the deltopectoral groove area was infiltrated with 20 ml ropivacaine (7.5%) as local anesthetic. Patients received anesthesia stand-by monitoring. If required, supplementary pain medication (such as metamizole or piritramide) or sedatives (like remifentanil, which serves dual roles as a sedative and analgesic or propofol) were administered during the operation depending on individual patient’s request/need and anesthesiologist’s assessment.

Postoperatively, patients were observed for approximately 2 h in the outpatient surgical facility and were usually discharged home thereafter. In case of venous puncture, a post-surgical chest X-ray was carried out in order to exclude a pneumothorax. Monitoring was continued for potential postoperative complications such as surgical site infections or issues related to the port system’s placement or functionality during the follow-up.

### Experimental intervention

For the experimental group, the CE-certified software application HypnoVR (HypnoVR SAS, Lampertheim, France, www.hypnovr.io) together with a commercially available VR headset (PICO G2 4 K All-In-One headset, Pico Technology, Beijing, China) has been used.

Patients had the option to select a VR environment from the following options: “winter magic,” “forest,” “tropical beach,” “deep sea diving,” “space voyage,” and “four seasons.” Additionally, they could pick a musical atmosphere (options included “relaxation,” “serenity,” “lounge,” “symphony,” “soft guitar,” “Asian ambiance”) and an optional additional guiding voice (either male or female) offering breathing directives and immersing them in the narrative. The VR scene was designed to shift gradually, minimizing the risk of inducing motion sickness.

Given the persistent requirement for verbal communication during the procedure, headphones were not employed. Instead, the VR headset’s built-in speakers delivered the musical atmosphere and vocal instructions.

The use of the VR setup aimed to create a calming and immersive environment, potentially reducing patient anxiety and discomfort. The VR experience was designed to be patient-centered, allowing for its discontinuation upon any sign of discomfort or at the patient’s request. Additionally, if patients experienced specific symptoms such as dizziness, nausea, or visual abnormalities, the VR would be immediately halted to ensure patient well-being.

### Data capture and trial endpoints

In this investigation, a comprehensive set of baseline and demographic data was collected for every participant, including their birth year, gender identity (female, male, or diverse), height, weight, body mass index, usage of glasses or contact lenses, classification under the American Society of Anesthesiologists system, underlying disease that necessitated port implantation, reasons for the implantation, any history of port implantations along with their count, pertinent comorbidities, and medical history including an assessment using the updated Charlson Comorbidity Index, covering a variety of health conditions (Quan et al. 2011).

Data collection on the day of surgery encompassed the following range of parameters: responses from a preoperative questionnaire and perioperative pain levels ascertained through the numerical rating scale (NRS). Participants completed a survey at three different points: before the operation, right after the operation, and prior to leaving the hospital, approximately 2 h after surgery. This survey gauged the patient’s current pain intensity using a numeric rating scale (NRS), where 0 signifies no pain and 10 represents the most severe pain imaginable. It also incorporated the six-item State-Trait Anxiety Inventory (STAI-6) to evaluate stress and anxiety during the perioperative period. STAI scores are commonly classified as “*no or low anxiety*” (20–37), “*moderate anxiety*” (38–44), and “*high anxiety*” (45–80) (Kayikcioglu et al. 2017; Nigussie et al. 2014).

Beyond the NRS and STAI-6, the pre-discharge survey inquired about symptoms of VR sickness like dizziness, nausea, vomiting, headaches, and fatigue. It also evaluated how patients tolerated the procedure (Likert scale: 1 = highly tolerable–5 = not at all tolerable) and their satisfaction level regarding the operation (Likert scale: 1 = highly satisfied–5 = extremely unsatisfied).

Furthermore, the surgeon’s contentment with the operation was also gauged (Likert scale: 1 = very pleased–5 = extremely displeased).

Other data captured included duration of surgery, the finally chosen technique for implantation, use of sonography-guided puncture, occurrence of any complications during the operation, types and quantities of perioperative analgesia, sedation, and local anesthetic administered, responses from an immediate postoperative questionnaire, time of patient discharge, the period from the end of surgery to discharge, a questionnaire completed prior to discharge, and any complications that arose postoperatively until the time of discharge.

The study meticulously documented and classified postoperative complications according to the Clavien-Dindo system (Dindo et al. 2004). The types of complications monitored included surgical site infections, incidents of postoperative bleeding or hematoma, thrombosis, cases of pneumothorax or hemothorax, issues with the port system such as dislocation or malfunction, catheter-related sepsis, and occurrences of nerve palsy.

For those patients assigned to the intervention group, information about their prior experiences with virtual reality (VR) was recorded. Further details noted included the chosen VR scenario, the hypnotic voice and musical background used, and whether the VR procedure was terminated prematurely, before the end of surgery.

Finally, a follow-up was conducted on the 30th day after the operation, wherein patients were contacted via telephone to evaluate any postoperative complications that might have developed following their discharge.

### Randomization

Randomization was performed preoperatively using the REDCap (Research Electronic Data Capture) randomization module, either 1 day before or on the day of surgery. The randomization module is fully integrated within the REDCap system, meaning researchers can seamlessly randomize participants based on data already entered into REDCap, ensuring consistency and efficiency in the study process. The module automates the process of random assignment of participants into different study arms. This automation ensures that the randomization process is unbiased and adheres to the predetermined allocation ratio (Harris et al. 2009). A computer-generated, balanced permuted block randomization sequence was used for randomization.

## Statistical analyses

In this pilot trial, all analyses were conducted in an exploratory manner, primarily focusing on estimation of standardized effect sizes and confidence intervals (CIs) for the purpose of sample size calculation for a potential subsequent confirmatory trial. Since the current trial was a pilot trial without formal sample size calculation, there was no designated primary outcome. Instead, the terms “main outcome” and “further outcome” parameters were used.

The final analysis included all patients treated with any of the trial interventions. The primary analysis strategy employed was an intention-to-treat analysis, analyzing patients in the groups to which they were randomized. A per-protocol analysis was conducted as a secondary analysis. Statistical evaluations included the empirical distribution of all endpoints. For continuous variables and scores, mean, standard deviation, and quartiles were calculated, while for categorical data, absolute and relative frequencies were determined. 95% CIs were computed for these measures. Descriptive *p* values from statistical tests were also provided, including the Fisher’s exact test for categorical variables and Welch’s two-sample *t*-test for continuous variables, along with associated 95% CIs for comparing treatment groups. Statistical graphics (e.g., boxplots) were utilized for visualizing findings where appropriate.

The homogeneity of the treatment groups was assessed by comparing demographic data and baseline values.

## Results

**Fig. 1:**
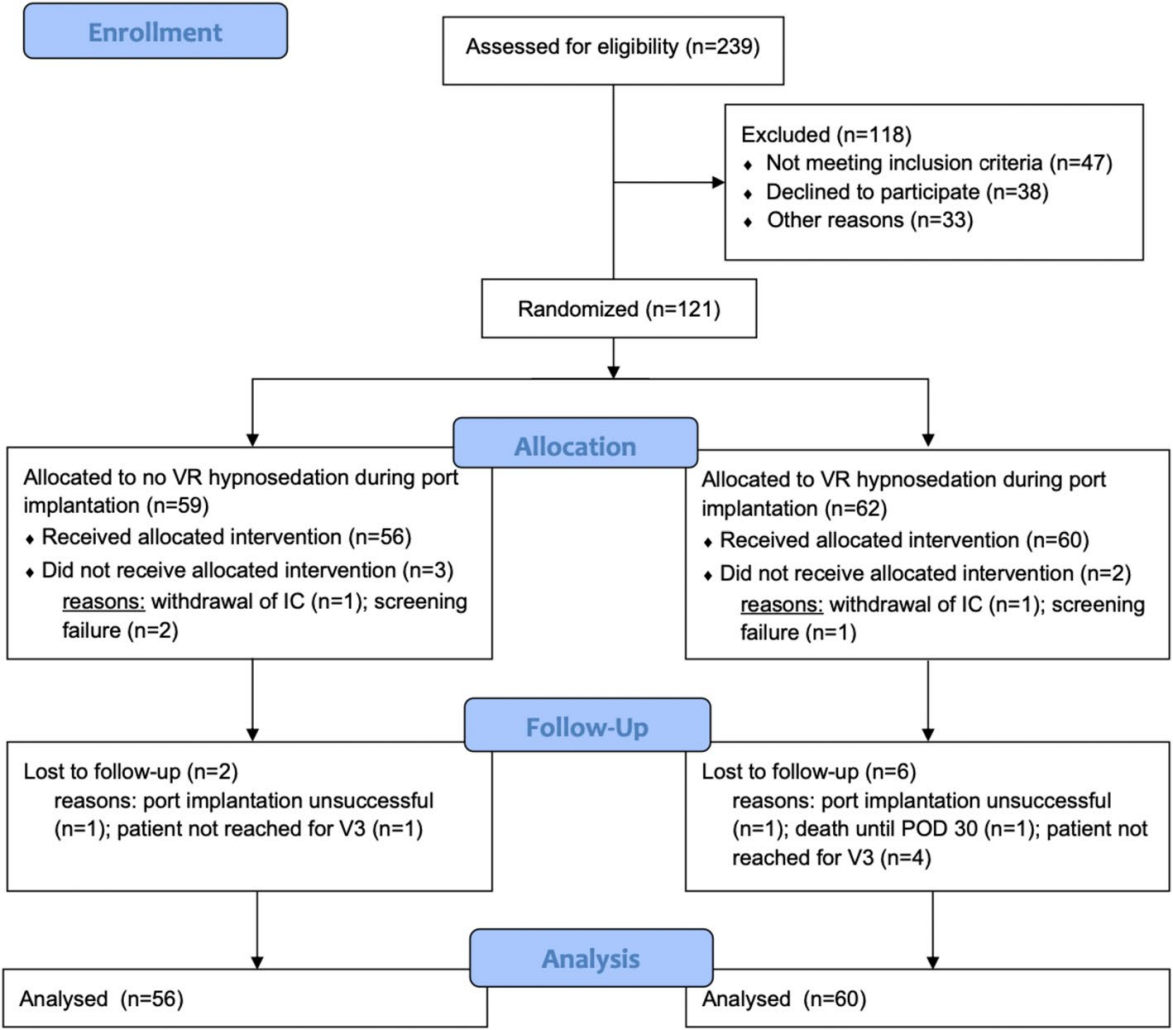
VIP Trial flow chart

A total of 121 patients scheduled for elective port implantation under LA were randomized within the trial between January 2022 and August 2023. However, two patients in the VR group and three patients in the no VR group were post-randomization drop-outs. Figure 1 depicts the trial flow of patients with reasons for exclusion at each stage. Finally, 60 patients in the VR group and 56 in the no VR group were analyzed. Demographic parameters and baseline data of the patients are displayed in Table 1. In total 8 (6.9%) of the patients were lost to follow-up during the 30-day period, but data from the day of surgery were available in all of these patients. Within these 8 patients, 2 (1.7%) port catheters could not be implanted and therefore surgery was terminated early. One (0.8%) patient died 17 days after port implantation due to disease progression.

**Table 1:** Demographic parameters and baseline data

Demographics	No VR (*n* = 56)	VR (*n* = 60)	Total (*n* = 116)
Age in years ± SD^a^	63 ± 12	62 ± 13.6	62 ± 12.6
Gender			
Male	43 (76.8%)	35 (58.3%)	78 (67.2%)
Female	13 (23.2%)	25 (41.7%)	38 (32.8%)
BMI in kg/m^2^ ± SD	25.3 ± 5.5	25.7 ± 6.1	25.5 ± 5.8
ASA^b^ category			
I	1 (1.8%)	0 (0%)	1 (0.9%)
II	15 (26.8%)	11 (18.3%)	26 (22.4%)
III	40 (71.4%)	49 (81.7%)	89 (76.7%)
Previous port implantations	7 (12.5%)	6 (10%)	13 (11.2%)
Disease			
Pancreatic carcinoma	10 (16.7%)	9 (15.0%)	19 (16.4%)
Esophageal carcinoma	8 (13.3%)	7 (11.7%)	15 (12.9%)
Gastric carcinoma	6 (10.0%)	4 (6.7%)	10 (8.6%)
Colorectal carcinoma	14 (23.3%)	12 (20.0%)	26 (22.4%)
Hepatocellular carcinoma	1 (1.7%)	1 (1.7%)	2 (1.7%)
Cholangiocellular carcinoma	2 (3.3%)	3 (5.0%)	5 (4.3%)
Other	15 (25.0%)	24 (40.0%)	39 (33.6%)
Charlson Comorbidity Index	3.2 ± 1.8	3.2 ± 1.8	3.2 ± 1.8
Implantation technique			
Venae section of cephalic vein	50 (89.3%)	52 (86.7%)	102 (87.9%)
Puncture of subclavian vein	4 (7.1%)	6 (10.0%)	10 (8.6%)
Puncture of internal jugular vein	1 (1.8%)	1 (1.7%)	2 (1.7%)

^a^*SD* Standard deviation

^b^*ASA* American Society of Anesthesiologists

Table 2 provides details on the selection of VR environments, voice preferences, and musical atmospheres in the interventional group. The “forest walk” environment was selected in 20 cases (33.3%), followed by the VR environment “tropical beach” in 15 cases (25.0%) and “deep sea diving” in 12 cases (20.0%). Other VR environments such as “four seasons,” “winter magic,” and “space voyage” were chosen in 5 (8.3%), 5 (8.3%), and 3 cases (5.0%), respectively. The majority of patients preferred a female voice, chosen in 36 cases (60.0%). Regarding the musical atmosphere, “relaxation” was the most frequently selected, in 44 cases (73.3%), followed by “serenity” in 7 cases (11.7%), “symphony” in 5 cases (8.3%), “soft guitar” in 3 cases (5.0%), and “lounge” in 1 case (1.7%).

**Table 2:** Details of VR hypnosedation

VR details (patient’s choice)	VR (*n* = 60)
VR environment	
Forest	20 (33.3%)
Tropical beach	15 (25.0%)
Deep sea diving	12 (20.0%)
Four seasons	5 (8.3%)
Winter magic	5 (8.3%)
Space voyage	3 (5.0%)
Voice	
Female	36 (60.0%)
Male	24 (40.0%)
Musical atmosphere	
Relaxation	44 (73.3%)
Serenity	7 (11.7%)
Symphony	5 (8.3%)
Soft guitar	3 (5.0%)
Lounge	1 (1.7%)

**Fig. 2:**
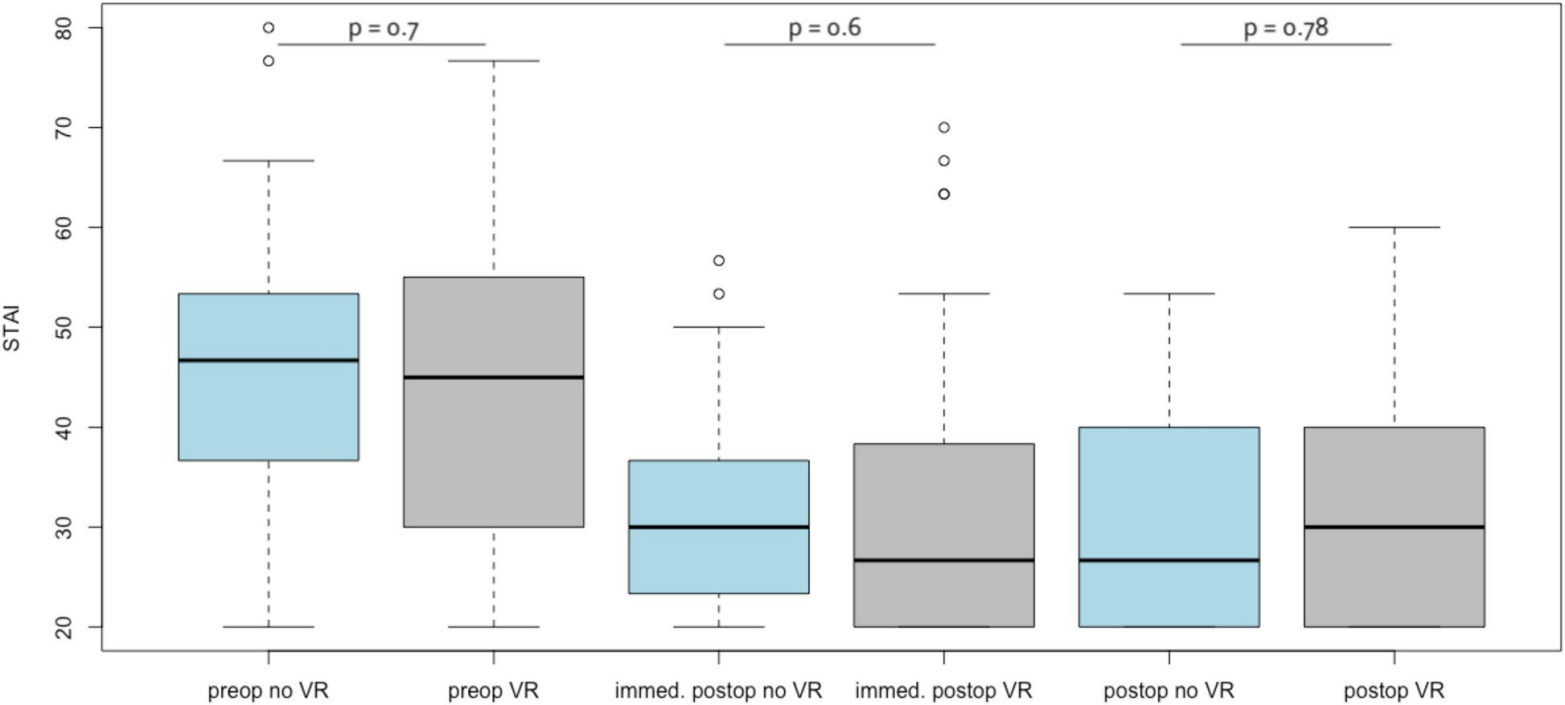
Boxplot diagram displaying STAI score preoperative (*p* = 0.7), immediately postoperative (*p* = 0.6), and before discharge (*p* = 0.78) in the no VR and VR group

**Fig. 3:**
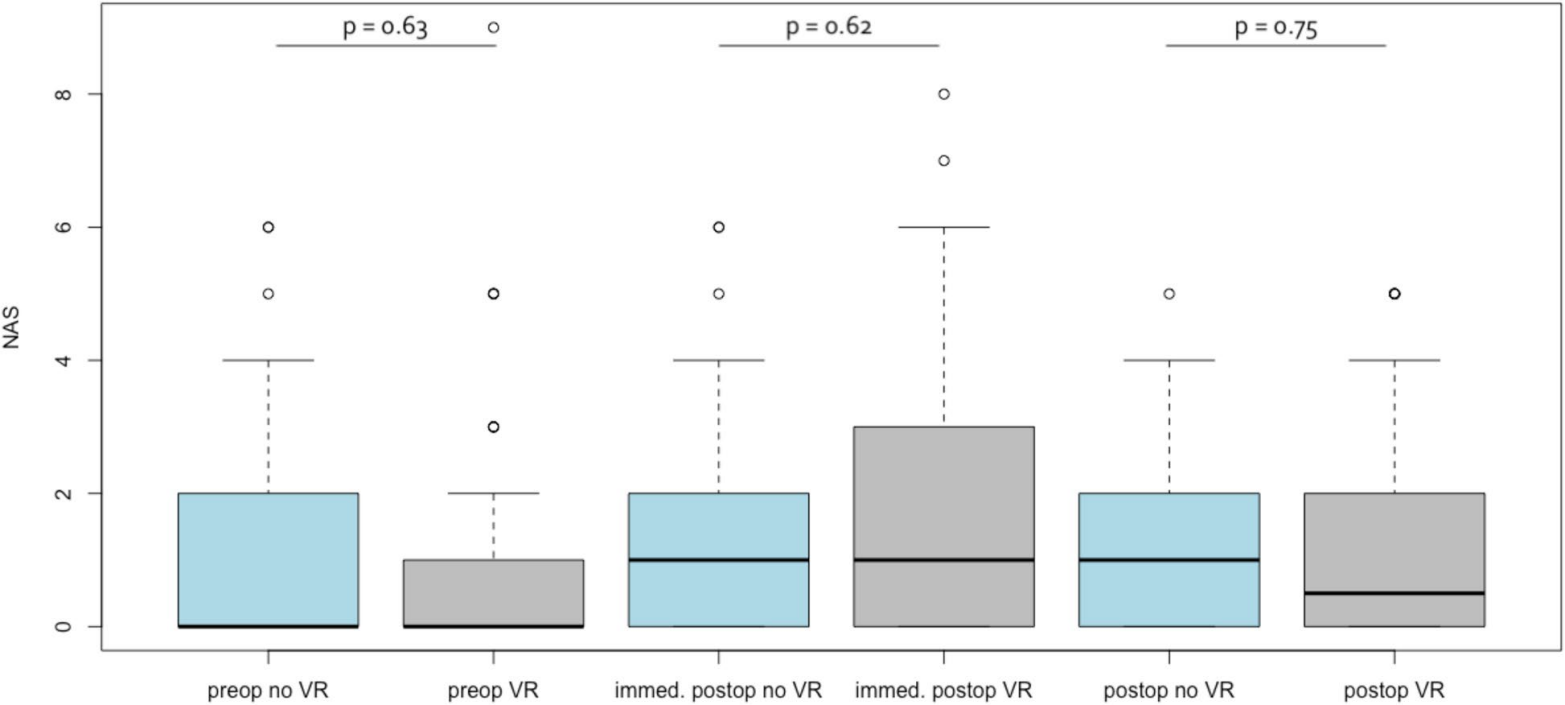
Boxplot diagram displaying NAS score preoperative (*p* = 0.63), immediately postoperative (*p* = 0.62), and before discharge (*p* = 0.75) in the no VR and VR group

Perioperative pain did not differ significantly at any point of time. The individual values for the different assessments are provided in Table 3. The perioperative anxiety level assessed by the STAI-6 was in the “moderate” range preoperatively and dropped to a “low” range postoperatively in both groups. However, there were also no statistically significant differences between the groups (Table 4). In addition, Figs. 2 and 3 display the perioperative course of pain and anxiety.

**Table 3:** Analgesic and sedative drug administration

Drug	No VR (*n* = 56)	VR (*n* = 60)	*p*-value
Novaminsulfon			
Yes	4 (7.1%)	5 (8.3%)	1.00
No	52 (92.9%)	55 (91.7%)	
Dose in mg^a^ (mean ± SD^b^)	1250 ± 500	900 ± 224	0.26
Ropivacain			
Yes	39 (69.6%)	45 (75.0%)	0.54
No	17 (30.4%)	15 (25.0%)	
Dose in mg (mean ± SD)	141 ± 49	141 ± 44	0.93
Propofol			
Yes	13 (23.2%)	10 (16.7%)	0.49
No	43 (76.8%)	50 (83.3%)	
Dose in mg (mean ± SD)	89.6 ± 54.2	75.4 ± 57.0	0.55
Midazolam			
Yes	4 (7.1%)	2 (3.3%)	0.43
No	52 (92.9%)	58 (96.7%)	
Dose in mg (mean ± SD)	2.6 ± 0.8	2.5 ± 0.7	0.86
Remifentanil			
Yes	7 (12.5%)	5 (8.3%)	0.55
No	49 (87.5%)	55 (91.7%)	
Dose in mg (mean ± SD)	200 ± 133	100 ± 66	0.12

^a^*mg* milligram

^b^*SD* standard deviation

**Table 4:** Peri- and postoperative metrics

Comparison	No VR Group (*n* = 56)	VR Group (*n* = 60)	*P *value
Preoperative Pain (Mean ± SD^a^)	1 ± 1.59	0.85 ± 1.79	0.63
Immediately Postoperative Pain (Mean ± SD)	1.43 ± 1.63	1.6 ± 2.05	0.62
Pain before discharge (Mean ± SD)	1.33 ± 1.31	1.24 ± 1.67	0.75
Preoperative Anxiety Score (STAI 6) (Mean ± SD)	45.24 ± 12.95	44.22 ± 15.34	0.70
Immediately Postoperative Anxiety Score (STAI 6) (Mean ± SD)	30.65 ± 9.13	31.78 ± 13.34	0.60
Anxiety Score (STAI-6) before discharge (Mean ± SD)	31.63 ± 10.46	31.03 ± 11.46	0.78
Duration of Surgery (Minutes) (Mean ± SD)	38.36 ± 13.26	41.37 ± 18.89	0.32

^a^*SD* Standard deviation

In the no VR group, 24 of 56 (42.8%) patients needed any type of sedative drugs, whereas in the VR group only 19 of 60 (31.7%) needed any type of sedative drugs. However, this difference was not statistically different (*p* = 0.17). The analgesic and sedative drugs administered and their dose during port implantation are displayed for both groups in Table 4. There were no relevant differences in postoperative symptoms of VR sickness between the study groups (Table 5).

**Table 5:** Symptoms of perioperative VR sickness

Symptom	No VR (*n* = 51)	VR (*n* = 54)	*p*-value
Nausea			
Yes	2 (3.9%)	0 (0%)	0.23
No	49 (96.1%)	54 (100%)	
Vomiting			
Yes	0 (0%)	0 (0%)	1.00
No	51 (100%)	54 (100%)	
Dizziness			
Yes	4 (7.8%)	1 (1.9%)	0.20
No	47 (92.2%)	53 (98.1%)	
Headache			
Yes	3 (5.9%)	4 (7.4%)	1.00
No	48 (94.1%)	50 (92.6%)	
Fatigue			
Yes	10 (19.6%)	11 (20.4%)	1.00
No	41 (80.4%)	43 (79.6%)	

The tolerance of the procedure from a patient’s perspective was not different between the two groups with a mean of 1.63 (± 0.78) in the no VR group and 1.41 (± 0.66) in the VR group (*p* = 0.12). Similarly, the satisfaction with the procedure differed neither from the patient’s (no VR: 1.47 ± 0.70 vs. VR: 1.41 ± 0.63; *p* = 0.63) nor from the surgeon’s perspective (no VR: 1.57 ± 0.82 vs. VR: 1.61 ± 1.09; *p* = 0.80). At 30 days after surgery, pain in the area of the port implantation was present in 4 out of 53 (7.5%) of patients in the no VR group and in 4 out of 54 (7.4%) of the VR group (*p* = 1.00). Forty-one of 53 (77.4%) patients in the no VR group and 42 of 53 (79.2%) patients in the VR group would have been hypothetically willing to undergo port implantation again under the same conditions (*p* = 1.00).

There was only one postoperative complication on the day of surgery before discharge in terms of a port misplacement detected in the postoperative X-ray in the VR group, where the tip of the port catheter was placed in the internal thoracic vein instead of the superior vena cava, requiring correction of the catheter placement. There was no complication on the day of surgery in the no VR group. Until postoperative day 30, 3 out of 53 (5.7%) patients in the no VR group experienced a postoperative complication and 7 out of 55 (12.7%) in the VR group (*p* = 0.32). A listing of postoperative complications is displayed in Table 6.

**Table 6:** Postoperative complications and Clavien-Dindo classifications

**Postoperative Complications**	**No VR (*n* = 56)**	**CD**^a^	**VR (*n* = 60)**	**CD**
Thrombosis	0 (0%)		2 (3.3%)	II
Port infection	1 (1.8%)	IIIA	0 (0%)	
Port misplacement	0 (0%)	IIIA	1 (1.7%)	
Surgical site infection	1 (1.8%)	II	0 (0%)	
Hematoma	0 (0%)		1 (1.7%)	I
Pleural effusion	0 (0%)		1 (1.7%)	IIIA
Other	1 (1.8%)	I	2 (3.3%)	I,V

^a^*CD* Clavien-Dindo Classifications

## Discussion

The current pilot trial on VR hypnosedation during port implantation shows that the application is feasible and safe. However, in the limited sample size of the current trial, there were no statistically significant differences in perioperative pain and anxiety. Nonetheless, we found some notable differences in the need for sedation during port implantation with 42.8% of patients in the no VR group and 31.7% in the VR group needing additional sedative drugs. To confirm this finding in a subsequent randomized controlled superiority trial, approximately 300 patients per group would be needed. Furthermore, we detected a reduced dose of the administered sedative drugs, especially remifentanil, in the VR group. While not statistically significant, the almost 50% reduction is noteworthy. Even though this finding is not statistically significant, it suggests that even if pain scores remained similar, the overall patient comfort might have been improved in the VR group, leading to decreased requirements for analgesic or anxiolytic agents. When considering novaminsulfon and propofol, the differences between the groups were not as pronounced; however, both were less administered in the VR group as well.

Regarding the patient-reported outcome parameters, the STAI-6 levels did not differ significantly in both groups at any point of time. However, there was a relevant drop from a preoperative moderate anxiety level to postoperative low anxiety levels in both groups. The satisfaction with and the tolerability of the procedure from a patient’s perspective did not differ significantly.

Comparative studies have shown mixed results regarding VR’s impact on pain and anxiety during surgeries. For instance, one study found lower pain scores in patients undergoing hand surgery with local anesthesia (LA) when exposed to VR (Garrett et al. 2018), while another study found no significant difference in pain perception or anxiety levels between the VR and non-VR groups for patients undergoing knee arthroscopy (Yang et al. 2019). Another study observed that males using VR during rigid cystoscopy experienced less pain compared to the no VR group, but such a reduction was not seen in females (Łuczak et al. 2021).

However, another recent study by Huang et al. aimed to assess the impact of VR on the sedation requirements of patients undergoing joint replacement surgery, conducting a randomized control trial involving 50 patients, where one group received VR along with propofol patient-controlled sedation. There were no significant differences in the patterns of propofol use over time between the two groups (*p* = 0.90) (Huang et al. 2020).

In contrast to our trial, a recent similar randomized controlled trial from Turkey assessing VR distraction during port implantation found statistically significant differences in postoperative pain and anxiety. The conflicting results might have different reasons: first, the VR intervention was not the same and different interventions might lead to different results; second, in the Turkish trial, the postoperative pain scores were substantially higher than in our trial and patients that received any analgesics were excluded from the trial; third, the anxiety levels decreases substantially in both groups of our trial, whereas for unknown reasons they even increased in the control group of the Turkish trial (Menekli et al. 2022).

Mechanistically, VR hypnosedation may reduce pain and anxiety through distraction, which diverts attention from surgical stimuli, and immersion, which fosters a sense of presence in a virtual environment, reducing awareness of the real-world procedure (Li et al. 2011; Wismeijer and Vingerhoets 2005). Hypnosis can further enhance relaxation, reducing the emotional and cognitive focus on pain. Neurobiologically, VR and hypnosis may modulate brain activity in areas responsible for pain processing (e.g., anterior cingulate cortex and somatosensory cortex) and enhance parasympathetic activation, thereby reducing stress (Patterson et al. 2006; Gupta et al. 2018). These mechanisms provide context for the potential efficacy of the intervention, despite the lack of statistically significant findings in the current study.

However, given the mixed results from various studies, including this one, the effectiveness of VR interventions in clinical settings remains uncertain. Factors such as the type of surgery, patient expectations, the quality and immersion of the VR environment, and adherence to standard anesthesiological protocols could potentially influence the outcomes of VR usage. Nonetheless, we believe that the following subgroup of patients might benefit from VR usage: patients with high preoperative anxiety. Given the trend observed in our study towards reduced sedative requirements in the VR group, patients who present with high levels of preoperative anxiety might experience greater comfort and reduced need for additional medication when using VR. This group may benefit from the immersive and distracting nature of VR, which could help alleviate anxiety and improve overall patient experience. Another subgroup includes patients with lower pain thresholds or sensitivity, since individuals who are more sensitive to pain or who have had previous negative experiences with medical procedures may find VR to be an effective tool for distraction and relaxation, potentially reducing their perception of pain and the need for analgesics. Additionally, we consider patients undergoing minor procedures with local anesthesia that VR could be particularly useful for procedures like port implantation, where the patient is conscious and may benefit from distraction to reduce discomfort and anxiety. The immersive nature of VR might be less effective in more complex or longer surgeries where deeper sedation is required. Also, patients with preference for non-pharmacological interventions, as some patients prefer to avoid medications, when possible, due to concerns about side effects or personal beliefs. For these patients, VR could serve as an appealing alternative to pharmacological sedation or analgesia.

There are some limitations that have to be considered in the interpretation of this trial. First, the current study was a pilot trial with limited sample size and without formal sample size calculation. Thus, the results can only be considered as exploratory, forming the basis for future research on this topic. Additionally, the potential for gender bias must be addressed, as there was an imbalance in the gender distribution between the VR and non-VR groups. Notably, the non-VR group contained a higher proportion of male patients, which could have influenced the outcomes. Second, due to the clear differences in the interventions, it was not feasible to blind either patients or the treating physicians. We acknowledge that the lack of blinding in our trial may have introduced bias in patient-reported outcomes, particularly in subjective measures like pain and anxiety. Patients’ awareness of their group assignment could have influenced their reporting, potentially leading to either a placebo or nocebo effect. While we included objective measures where possible, the subjective nature of these assessments remains a limitation.

Future studies could address this by incorporating blinding or using objective physiological markers alongside self-reported outcomes to better assess the intervention’s efficacy.

Third, in both arms additional analgosedation was permitted as needed. This raises the question of whether the variations in sedative drug use were more a reflection of differences among anesthesia providers rather than the direct effect of the hypnosedation. Consequently, it is possible the anesthesiologists might have administered drugs based on standard protocols rather than adapting to dynamic patient feedback, given the lack of precise guidelines in this area. Future trials should be designed to specifically address and control for the influence of anesthesiologists’ actions. This should include a clear protocol for sedative administration and documentation, as well as stratifying results based on the level of sedation to better isolate the effect of the VR intervention itself.

Furthermore, it could be speculated that the effectiveness of the VR intervention might have been notably diminished by the ambient noise in the operating room, potentially serving as a significant distraction, especially since VR headphones were not utilized to facilitate communication. Future trials should allow for the operator or anesthesiologist to mute and unmute communication with the patient, as needed, which would allow for critical interaction without breaking the continuity of the hypnosedation effect.

Fourth, the study did not account for variability in underlying diseases, which could influence patient outcomes. Finally, the port implantations were conducted by different surgeons, usually resident surgeons accompanied by a senior surgeon, and therefore differences in surgical experience between the practitioners might have influenced the results. However, the purpose of this pilot trial was to reflect clinical reality and thus we did not change the practice at our hospital in this regard.

All in all, the study protocol could have been improved by minimizing some confounding factors, such as stricter control of anesthetic administration and more standardized procedures would have reduced variability.

In conclusion, this pilot trial suggests that VR hypnosedation might reduce the need for additional sedation, potentially through enhanced patient comfort or relaxation. VR hypnosedation might have the potential to reduce reliance on pharmacological interventions by offering a non-invasive alternative for managing perioperative pain and anxiety. This approach could be especially valuable in settings where minimizing drug use is a priority, such as in patients with contraindications to certain medications or those at risk of drug-related side effects. The lack of significant differences in perioperative pain or anxiety in this study, coupled with the inconsistent evidence from prior literature, suggests that future research should be more targeted with the need for larger, confirmatory trials to better establish the role of VR in perioperative care and to determine whether these trends translate into significant clinical benefits. If pursued, it should focus on identifying specific contexts or patient groups where VR interventions might be most beneficial and on exploring the customization VR environments tailored to individual patient preferences or even augmenting the VR experience with guided relaxation or meditation techniques for enhanced efficacy. Expanding our understanding of VR’s potential could ultimately lead to more comprehensive, patient-centered approaches in surgical care.

## Data Availability

The datasets used and analysed during the current study are available from the corresponding author on reasonable request.

## Data availability

No datasets were generated or analysed during the current study.
